# Understanding autism: structural and functional characterization of PTCHD1

**DOI:** 10.1063/4.0001144

**Published:** 2025-10-27

**Authors:** Adrian Goldman, Mimmu K Hiltunen, Orquidea Ribeiro, Natalia Riobo-del Galdo

**Affiliations:** 1 University of Helsinki, Helsinki, Uusimaa, Finland; 2 University of Leeds, Leeds, West Yorkshire, United Kingdom

## Abstract

Autism (MIM 209850) is a neurodevelopmental condition with heterogeneous aetiology, characterised by restricted interests, repetitive behaviour, and atypical social interaction. Many genes have been associated with autism, several of which encode for proteins involved in synaptic processes, but mutations in the X-linked patched-domain containing 1 (*PTCHD1*) gene (MIM:300828; Xp22.11) account for up to 1 % of cases of autism [1,2]. *PTCHD1* is expressed throughout the brain and other tissues, and its expression profile in the brain changes as development proceeds [3]. The PTCHD1 protein is synaptically localised in neurons, and mutations disrupt its expression, stability, glycosylation, and localisation [3]. While the exact functional role of PTCHD1 remains elusive, several findings implicate an essential role in developmental processes, the development of autism and intellectual disability [2]. PTCHD1 is a member of the Patched-domain containing protein family, which also includes the Hedgehog (Hh) pathway receptor Patched 1 PTCH1) and Niemann-Pick disease type C1 (NPC1). These proteins are structurally homologous and contain 12-13 transmembrane helices TM), a sterol sensing domain (SSD) composed of five TMs, a sterol sensing- like domain, and two ectodomains (ECDs). Full-length PTCHD1 is an 888-amino-acid, 12-pass transmembrane protein containing an SSD composed of TM 2-6 and a sterol sensing-like domain consisting of TM7- 12 followed by a C-terminal tail containing a PDZ-binding motif (ITTV). Additionally, two large ECDs are inserted between TM1 and 2 and TM7 and 8 (Figure 1). Cholesterol transporters are involved in fundamental cellular processes. Understanding the function of PTCHD1 could provide key information on the association of PTCHD1 with autism. We have gained insight into the unique features of PTCHD1 by combining *in vitro* and *in silico* methods, using an AlphaFold2 generated structure. **Structure prediction reveals conserved cholesterol-binding elements, and a click-cholesterol assay confirms PTCHD1 binds cholesterol** In our recent publication, we showed the AlphaFold predicted PTCHD1 is structurally homologous to several cholesterol transporters belonging to the Patched domain-containing family, including Dispatched 1 (DISP1), NPC1, and PTCH1 (Figure 1) [4]. While the overall sequence conservation between these proteins is low, sequence alignment revealed a higher degree of conservation in the SSD (17 %, 18 %, and 23 % identity with DISP1, NPC1 and PTCH1, respectively) than in the entire sequences, indicating a shared function in cholesterol binding/ transport. To investigate this possibility, we performed a cholesterol click-reaction assay on purified PTCHD1 elution fractions. Purified PTCHD1 was cross- linked with a PhotoClick cholesterol with UV light and then labelled by a ‘click-reaction’, followed by analysis by SDS-PAGE and imaging. The results show that PTCHD1 can bind cholesterol (Figure 2A): strong bands of ∼130 kDa and ∼100 kDa, corresponding to the approximate molecular weights of fully glycosylated and not glycosylated PTCHD1, are present in all lanes of treated PTCHD1 solubilised in varying amounts of GDN. **Docking indicates PTCHD1 can bind cholesterol similarly to PTCH1** Superimposing our PTCHD1 model onto PTCH1 showed that the SSDs are nearly identical in structure [2]. To further explore the similarities in cholesterol binding between PTCHD1 and PTCH1, we performed docking analysis using AutoDock Vina. The full SSD of PTCHD1 (TM2-6 of AF- Q96NR3) or PTCH1 (TM2-6 of AF- Q13635), used as a control to validate the docking method, was defined as the docking site. We compared cholesterol binding by aligning docked cholesterols to the cryo-EM structure of the PTCH1 (6RMG) cholesterol-binding sites: site 1 is within the outer leaflet pocket, site 5 is in the inner leaflet portion of the SSD, and site 4 is on the opposite side of the SSD, adjacent to TM1 (Figure 2B). Cholesterol docked to three sites on the PTCHD1-SSD with estimated binding energies ranging from -8.7 kcal/mol to -7.2 kcal/mol. The predicted cholesterol-binding positions in PTCHD1 directly overlap with cholesterols in the PTCH1 cryo-EM structure: 25/27 cholesterol binding positions are in site 1 between TM3, TM4, and TM6. Additionally, two cholesterol binding positions are near site 5 in the putative inner leaflet portion of the SSD, adjacent to TM3 (Figure 2B). In the case of PTCH1-SSD, the estimated binding energy is between -8.4 kcal/mol and -7.3 kcal/mol. In addition, 23 docked cholesterol- binding positions overlap with binding site 1 and four near site 4 (Figure 2B). The similar binding of cholesterol is unsurprising due to the SSDs of PTCHD1 and PTCH1 sharing nearly 23 % identity.

Preliminary data suggests autism-linked variants disrupt cholesterol transport

To further elucidate the interaction of PTCHD1 with cholesterol, and investigate how this links to autism, we have employed a fluorescence-based *in vitro* cholesterol transport assay in HEK293T. Our preliminary data indicates PTCHD1 overexpression increases cholesterol export by ∼10% compared to a negative control (cells transfected with pcDNA3), whereas some autism-linked variants of PTCHD1 do not. This observation is supported by preliminary docking results, which indicate autism-linked mutations in the PTCHD1 ECDs influence how they interact with cholesterol.

Structural analysis and complex prediction show PTCHD1 is unlikely to bind Shh

The structurally similar protein PTCH1 uses cholesterol to modulate Smoothened activity in canonical Hh signalling. However, it is not clear if PTCHD1 shares this function [3,5]. We used AlphaFold2 to predict the complex formation of N-terminal Shh (ShhN, res 23-197) with the ectodomains (ECDs) of PTCHD1 and, as a positive control, the ECDs of PTCH1. Alignment of the predicted PTCHD1 ECD1 and ECD2 onto the full AlphaFold2 predicted structure (AF-Q96NR3) indicates the conformation of each ECD and their interaction has been predicted well (RMSD 0.961 Å). Furthermore, the predicted PTCH1:Shh structure aligns well with a complex solved by cryo-EM (RMSD 1.606Å with 6DMY) and contains 6/10 of the hydrogen bonds found in the complex indicating AlphaFold2 can predict Shh binding [4]. ShhN is predicted to bind to a β- hairpin and an α-helix on ECD2 of PTCHD1 (Figure 2C, left). However, the predicted aligned error is very high between ShhN and the ECDs for PTCHD1, suggesting ShhN is unlikely to form a complex with PTCHD1 ECDs. The low-likelihood of complex formation between PTCHD1 and ShhN is consistent with our in vitro data:a GLI luciferase assay in Ptch1-/- mouse embryonic fibroblasts showed transient expression of PTCHD1 does not reduce GLI-luciferase activity compared to the empty plasmid (pcDNA3.1+ control), whereas transfecting cells with PTCH1 inhibited GLI-luciferase activity by ∼65 %. Furthermore, a LigandTracer™ experiment showed there is no binding of Shh to PTCHD1 expressed in Sf9 cells.

whereas transfecting cells with PTCH1 inhibited GLI-luciferase activity by ∼65 %. Furthermore, a LigandTracer™ experiment showed there is no binding of Shh to PTCHD1 expressed in Sf9 cells. In-depth structural analysis suggests the lack of binding is due to the absence of ECD loops, which form a negatively charged pocket and mediate binding in PTCH1 (Figure 2C, right) [4]. The inability of PTCHD1 to bind Shh and to inhibit GLI transcription in MEFs argues against the possibility of acting as a Hh receptor, despite sharing structural features with PTCH1. The absence of Hh-related phenotypes in PTCHD1- deficient mice also supports our conclusion [2]. Although our *in vitro* experiments and *in silico* structural analysis suggest PTCHD1 does not bind Shh nor inhibit Hh signalling, we cannot rule out that PTCHD1 influences the Hh pathway in a noncanonical fashion and/or in a cell type-specific manner. In conclusion, our findings support the idea that PTCHD1 is functionally unique and participates in fundamental cellular processes, different to PTCH1 and NPC1. First, we showed that PTCHD1 is not a PTCH1 functional homolog. Second, we showed that PTCHD1 binds cholesterol but not Shh and cannot inhibit canonical Hh signalling. Third, we identified key features in the PTCHD1 sequence and structure that distinguish it from other Patched-domain containing family members.
